# Driving rural industry revitalization in the digital economy era: Exploring strategies and pathways in China

**DOI:** 10.1371/journal.pone.0292241

**Published:** 2023-09-28

**Authors:** Gongli Luo, Yu Yang, Lu Wang

**Affiliations:** College of Economics and Management, Shandong University of Science and Technology, Qingdao, China; Shenzhen University, CHINA

## Abstract

In the context of the digital economy, achieving rural industrial revitalization in China hinges on digitization. This study delves into the synergistic mechanisms of diverse factors that contribute to Rural Industrial Revitalization across three dimensions: technology, organization, and environment. To investigate these mechanisms, a combination of the necessary condition analysis method and the fuzzy set qualitative comparative analysis method is employed. The research findings indicate that no necessary conditions exist for achieving high-level rural industrial revitalization. However, digital infrastructure and the digital financial environment have a universally significant impact. The study identifies four distinct pathways driving high-level rural industrial revitalization: digitaldriven, digital-government-talentdriven, digital-enterprisedriven, and digital-enterprise-talentdriven. Furthermore, significant variations exist in the driving pathways for rural industrial revitalization among the eastern, central, and western regions of China. By unveiling the multifaceted mechanisms underpinning the revitalization of rural industries, this research provides valuable practical insights for the future development of rural industries in China.

## 1. Introduction

Presently, China grapples with a widening urban-rural development disparity, marked by substantial challenges and decline in rural areas. To address these concerns regarding agriculture, rural regions, and farmers ("San Nong" in Chinese), China has implemented the rural revitalization strategy. Recognizing digital infrastructure and digital finance as pivotal and dynamic elements that align with the progress of traditional industries, China actively merges the digital economy with the real economy. This convergence has emerged as a pivotal propeller for China’s pursuit of high-quality economic development [1]. The China Academy of Information and Communications Technology highlights that in 2022, China’s digital economy surged to 50.2 trillion RMB, constituting 41.5% of the nation’s GDP. However, while industrial digitization accelerates across sectors, the agricultural domain lags behind, with a meager 8.9% penetration rate of the digital economy. In contrast to the levels of digitization witnessed in secondary and tertiary sectors, the application of digital technologies in agriculture remains relatively limited in terms of quality and depth [2]. Certain areas with lower levels of development experience slower progress in digital rural development, while remote regions face challenges in establishing a conducive environment for digital infrastructure and Digital financial environment(DFE) [3]. Additionally, government financial support is inadequate [4], and there is a scarcity of skilled professionals specializing in basic digital rural expertise [5]. Consequently, to address these challenges, China issued the "Digital Rural Development Strategy Outline" in 2019, aiming to vigorously promote digital rural construction and rural revitalization. By 2023, the China Central Government’s No. 1 Document delineated pivotal objectives for the comprehensive advancement of rural revitalization, underscoring the significance of intensified endeavors in promoting digital rural development.

In the realm of complex organizational governance, the "configurational perspective" has gained wide recognition as a means to comprehend the intricate causal complexity within organizations [6]. This perspective regards various influencing factors as interdependent, achieving their desired impact through diverse combinations. However, existing research often focuses on the influence of a single factor on rural industrial revitalization (RIR), examining linear relationships between them. This limited approach fails to provide an in-depth exploration of the reasons behind variations in RIR and neglects the analysis of core conditions and their configurations, thereby lacking a systematic revelation of the complex mechanisms of multi-factor interactions. As a result, future research should emphasize the analysis of configurations driving RIR, effectively differentiating the core conditions that influence it, and elucidating the intricate mechanisms through which different conditions impact RIR.

This paper adopts the Technology-Organization-Environment (TOE) framework and employs Fuzzy Set Qualitative Comparative Analysis (fsQCA) to elucidate the complex interplay of multiple conditions behind rural industrial revitalization in the context of the digital economy, addressing the limitations of previous research in explaining these issues. The contributions of this paper are as follows:

1. By adopting a "configurational perspective," the study shifts the focus of RIR research from singular perspectives, such as policy support and technological means, to a holistic view of the interplay between technology, organization, and environment.
2. The introduction of the fsQCA method enriches the methodological toolbox in the field of rural industrial development research. It provides a comprehensive perspective on the complex interactions and causal asymmetry among various conditions involved in its governance, offering a more integrated view.
3. In addition to conducting configurational analysis at the national level, the study also considers geographical and economic differences to analyze the diverse pathways of rural industrial development in China’s eastern, central, and western regions. This exploration is of significant importance for different regional governments in formulating more effective policies in the context of the digital economy.

## 2. Literature review and theoretical framework

### 2.1. Literature review

As the digital economy progresses, a plethora of scholars have delved into comprehensive investigations concerning the association between digital infrastructure and rural industries. In relation to the investigation of the correlation between e-commerce and the development of diverse rural industries, rural e-commerce assumes a prominent position as a crucial element within the realm of rural industries. It serves as a catalyst for the rejuvenation of the rural economy and the augmentation of farmers’ income [7, 8]. However, rural areas face challenges such as market information asymmetry and high transaction costs, as well as a digital divide between urban and rural areas, which limits the sales channels and sales volume of agricultural products [9, 10]. To address these issues, digital platforms can be built, allowing rural areas to establish their own e-commerce sales platforms online, thus breaking through geographical restrictions and expanding the market scope. Compared to urban areas, rural areas have advantages in labor and rent, which can not only increase employment opportunities but also narrow the urban-rural gap [11]. A study examining the online sales of rural fruits in China has revealed that the lack of digital infrastructure represents a primary impediment to the development of the e-commerce industry, indicating significant room for improvement within the Chinese rural e-commerce sector [12]. In the rural tourism industry, digital technology is increasingly being used to promote and advertise more accurately through social media, attracting more tourists [13]. Although tourism planning is complex due to difficulties in obtaining information, online booking, payment, smart tourism, network marketing, and other digital services provide convenience for tourists [14], prompting the rural tourism industry to further improve service quality and customer experience. Digital agricultural industry development is another area of focus [15], with literature primarily focusing on digital infrastructure such as information and communication technology [16], precision agriculture [17], smart agriculture [18], big data analysis [19], and the Internet of Things(IoT) [20]. Researchers are using artificial intelligence and big data analysis to transform traditional agriculture into a highly automated and data-intensive industry to solve agricultural production problems [21]. The application of the IoT in the agricultural management process can realize remote monitoring of agricultural systems, disease diagnosis, and improve various decision-making processes to achieve the rational use of resources [22]. For example, it can be used to monitor agricultural irrigation [23], pesticide spraying concentration, and fertilizer usage [24].

Digital inclusive finance, which possesses features such as high efficiency, inclusiveness, and low cost, is becoming a new trend in rural financial development. The utilization of digital finance technology can enhance the business processes and risk management capabilities of rural financial institutions, decrease operational costs, elevate service efficiency, and effectively stimulate the prosperity of rural industries [25]. Consequently, it can achieve rural economic revitalization and promote the sustainable development of rural inclusive finance. Digital inclusive finance has both macro and micro-level effects on the promotion of rural industrial development. At the macro level, the development of digital inclusive finance provides new impetus for the transformation and upgrading of the rural economy, accelerates the modernization process, and promotes the healthy development of the rural financial system. This has significant implications for promoting the high-quality development of China’s agricultural economy. From a micro perspective, digital inclusive finance offers comprehensive financial services such as financing, credit, insurance, and others, to rural industries, thereby narrowing the income gap between urban and rural areas and effectively promoting the rapid development of rural industries [26, 27]. Furthermore, the impact of digital finance on rural economies has gained significant attention in current research. Scholars posit that the adoption of digital inclusive finance can effectively mitigate the income disparity between urban and rural dwellers [28]. In their study, Grossman and Tarazi [29] discovered that digital inclusive finance facilitates income augmentation for farmers by enabling convenient payment mechanisms. Nevertheless, certain studies suggest that digital inclusive finance might inadvertently impede agricultural production for households, primarily due to an expanding disparity between the efficiency of non-agricultural economic activities and agricultural production [30].

In conclusion, the RIR is a multifaceted undertaking. Prior investigations have scrutinized the interplay between digital infrastructure, digital financial environment, and rural industries, underscoring the paramount significance of digital infrastructure and digital finance in the prevailing age of digitization. Nevertheless, the majority of studies have primarily focused on analyzing individual industries or specific factors, neglecting comprehensive research on the holistic impact of rural industries and lacking an in-depth exploration of attribution configurations and diverse driving mechanisms. Furthermore, existing empirical research has predominantly examined causal relationships characterized by symmetry, disregarding the synergistic effects among multiple conditions and asymmetrical causal analysis. Consequently, the phenomenon of differentiation in RIR has not been fully considered. Lastly, owing to the intricate and dynamic nature of RIR, it becomes apparent that alterations in a single factor often yield far-reaching consequences and interact with other factors, giving rise to interconnected outcomes.

### 2.2. Theoretical framework

The Technology-Organization-Environment (TOE) framework, originally proposed by Tornatizky and Fleischer in 1990, serves as a comprehensive analytical tool for technology governance [31]. This framework revolves around three primary dimensions. Firstly, the technology dimension centers on scrutinizing the technological attributes, application capabilities, and potential benefits within the organization [32]. Secondly, the organization dimension encompasses factors such as organizational size, scope, communication mechanisms, and resource reserves [33]. Lastly, the environment dimension takes into account external factors, including government regulations, policies, and market structure that influence the organization’s operations [34]. Since its inception, scholars have conducted robust empirical research guided by the TOE framework and have extensively applied it in the field of information systems, continually enriching the model’s content across diverse contexts [35].

**Fig 1:**
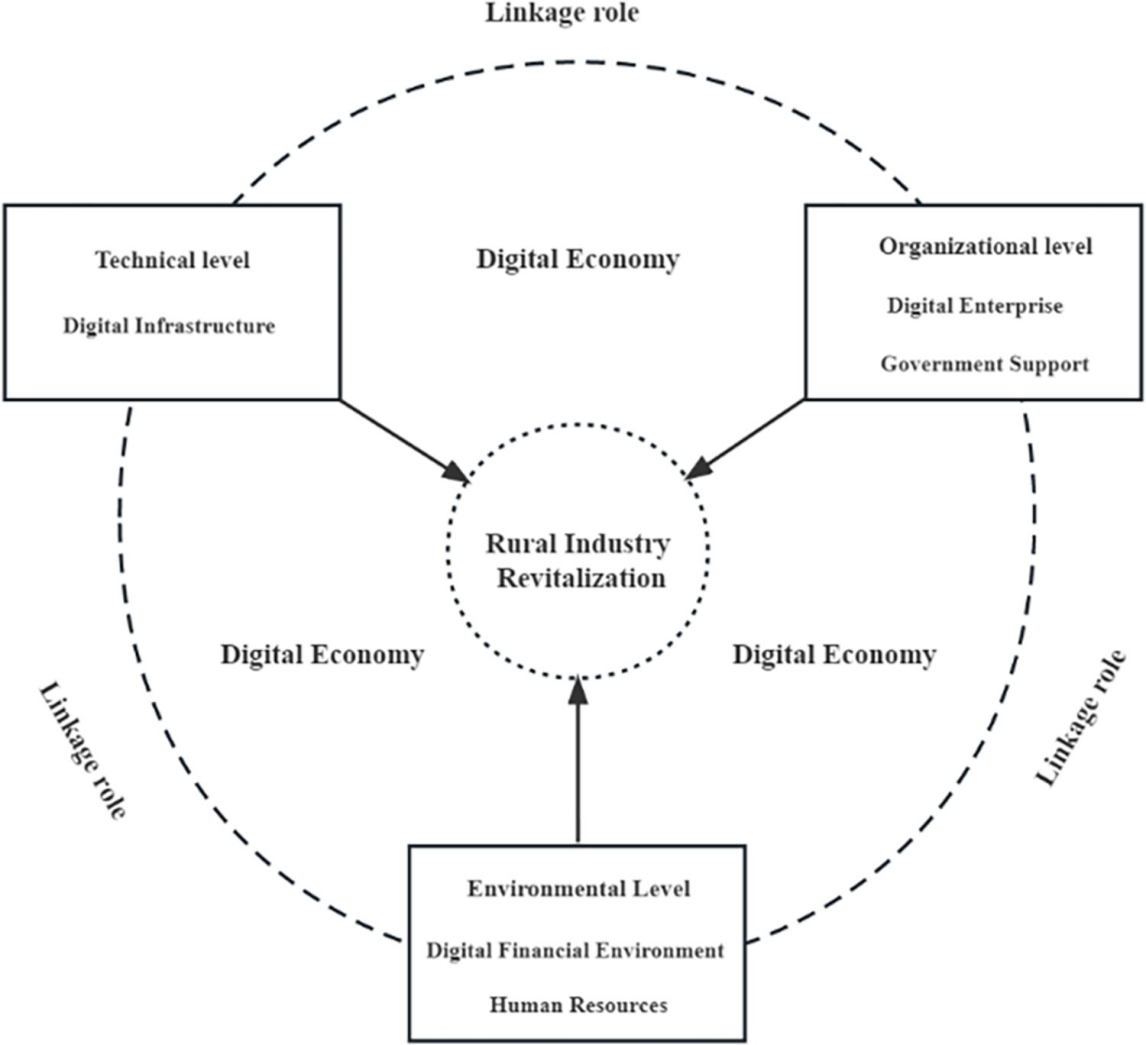
Analytical framework of rural industry revitalization.

The TOE theory is a versatile framework capable of encompassing multiple factors and adapting flexibly to various research questions and contexts. Due to its wide applicability, the TOE theory is not limited to use in e-government and e-open systems; it is also being adopted to explore the diffusion of innovative technologies within public organizations. For instance, Tan et al. utilized the TOE theory to analyze organizational behavior and identify the factors influencing the performance of local government websites, taking into consideration technological, organizational, and environmental conditions. Subsequently, they conducted fsQCA configurational analysis using a sample of 31 provincial-level government portal websites in China [36]. Therefore, the advantages of applying the TOE theory are highly applicable to the examination of RIR. Successful RIR necessitates continuous adjustments to strengthen social governance capabilities and adapt to the evolving demands of the times. To investigate the development of rural industrial revitalization comprehensively, it is essential to consider the interplay of technological, organizational, and environmental conditions. By employing the TOE theory in the research on RIR, it enables a deeper analysis of the key factors influencing the revitalization process, thereby fostering enhanced economic development and social progress in rural areas. This article utilizes the TOE analysis framework to integrate the context of rural industrial development in China with the backdrop of the digital economy, resulting in the establishment of a theoretical model framework for influencing RIR, as illustrated in Fig 1.

The initial determinant of RIR is the technological condition, encompassing digital infrastructure. The advancement of digital infrastructure, comprising communication infrastructure, computer infrastructure, data center infrastructure, cloud computing infrastructure, and other associated facilities, assumes a pivotal role in furnishing indispensable technological support for rural revitalization within the digital economy [34]. Through the utilization of digital infrastructure, rural industries can attain information sharing, data exchange, collaborative innovation, and online marketing, thereby fostering heightened productivity and economic gains. Moreover, digital infrastructure presents an avenue for the digital transformation of conventional rural industries, facilitating their rapid and extensive integration into the digital economy. Consequently, this integration enhances their core competitiveness and adaptability [37]. Therefore, as an essential component of the digital economy, digital infrastructure plays an irreplaceable role in RIR.

The second factor is the organizational condition, which comprises two sub-variables: digital enterprises and government support [38]. Both digital enterprises and government support are essential for RIR. Digital enterprises provide advanced digital technologies and business models, while government support offers policy guidance and financial assistance. The combination of these factors creates a robust support system for the development of rural industries. Digital enterprises enhance product quality and market competitiveness by integrating online and offline resources and optimizing the industrial chain [39]. In the field of digital agriculture, these enterprises construct agricultural IoT platforms to monitor and manage the complete production process, leading to significant improvements in agricultural productivity, output, and cost [40]. The government plays an active role in encouraging and guiding enterprises and individuals to participate in the development of rural industries through policies, regulations, financial support, and the optimization of the business environment. Furthermore, the government promotes the deep integration of rural industries with the digital economy.

The third factor is the environmental condition, consisting of two sub-variables: digital financial environment and human resources [41]. The digital financial environment is an essential condition for the RIR. It provides convenient financial services, such as small loans, insurance, and payment settlements, to rural industries through digital technology and financial means. This helps them integrate better into the digital economy and market system [42]. Moreover, digital rural development requires high-quality talent support. The talent reserve and flow in rural areas are crucial guarantees for the development of rural industries. In the digital economy era, talent with digital thinking and skills are necessary to support the digital transformation and development of rural industries. It is also essential to strengthen rural talent training and introduction to improve the competitiveness and innovation capability of rural industries [43].Therefore, the effective utilization of the digital financial environment and human resources is critical for the RIR.

In conclusion, the influencing factors of "RIR" are multifaceted and varied. By analyzing the three key elements: technology, organization, and environment, we have identified a strong logical connection among them. Firstly, digital infrastructure plays a vital role in providing organizations with essential services such as information, data, and communication, facilitating efficient sharing of information and resources within the organization. Simultaneously, enterprises can leverage technology to achieve digital transformation, while enhanced infrastructure enables the government to provide more targeted policy support. Secondly, technological advancements require supportive environmental conditions. The digital financial environment offers financial backing for technological development, while the human resources environment contributes high-quality talent for technological progress. It is evident that the advancement of technology relies on critical factors at the environmental level. Lastly, the development of digital enterprises necessitates a stable digital financial environment and an ample supply of human resources. Thus, improving environmental conditions ensures necessary prerequisites for the development of organizational conditions. In summary, the factors influencing RIR are not isolated or simplistic; instead, they form a systemic and intricate framework. The interplay among technology, organization, and environment generates interconnected relationships, propelling RIR in a configurational fashion.

## 3. Research methods

### 3.1. fsQCA and NCA research methods

The Fuzzy Set Qualitative Comparative Analysis (fsQCA) method is a multivariate statistical technique used to examine causal relationships [44]. It was developed by American sociologist Charles C. Ragin in 1987, with the aim of addressing complex social phenomena. This method is suitable for studies with large, medium, and small sample sizes, and is particularly advantageous for small sample studies. It is suitable for cases where it is difficult to obtain a large amount of continuous statistical data for time-series and panel data analysis. Unlike the use of simple linear regression methods in the past, the fsQCA method considers multiple factors as combined conditions to explore optimal paths. It can identify combinations with explanatory power and finer-grained causal mechanisms for complex phenomena. The diversity in the paths of achieving RIR in various regions of China suggests the existence of equivalent conditions, meaning different combinations of conditions lead to the same outcome. The Necessary Condition Analysis (NCA) method, developed by Professor Dul from the Rotterdam School of Management in the Netherlands in 2016, can effectively determine whether antecedent conditions are necessary for specific outcomes, which can provide effective supplementation for the necessity analysis of fsQCA. While the results of fsQCA after calibrating antecedent conditions are membership scores indicating the "yes or no" of necessary conditions, NCA can not only identify the necessary conditions for RIR but also determine the minimum level of necessary conditions by calculating the effect of each condition on the outcomes. Therefore, combining NCA and fsQCA can yield greater value [45].

### 3.2. Research samples and data sources

Sample selection for this study involved selecting 30 provinces of China (excluding Tibet and the Hong Kong, Macao, and Taiwan regions) as research cases, which meets the requirements for a small sample size in QCA research methods. The selection was made according to the principles of sufficient homogeneity and maximum heterogeneity within the population. The 30 selected provinces have significant differences in rural industry development, which ensures sufficient homogeneity of the case population and maximum heterogeneity within it.

The data sources and descriptive analysis are shown in Table 1.

**Table 1:** Data sources and descriptive analysis.

Conditional and result variables	Data sources	descriptive analysis of the dataset			
		Mean	Std. Dev.	Minimum	Maximum
Y: RIR	China Rural Statistics Yearbook, the National Bureau of Statistics and China Financial Statistics Yearbook.	0.30	0.14	0.06	0.63
X1: DI	China Statistical Yearbook and National Bureau of Statistics.	0.20	0.17	0.02	0.74
X2: DE	China Statistical Yearbook and provincial statistical yearbooks.	10.28	3.49	5.50	22.80
X3: GS	Business-related data from E-Commerce Reporting Statistics	6.43	6.56	2.43	30.84
X4: DFE	the Peking University Digital Financial Inclusion Database.	342.24	34.83	298.23	431.93
X5: HR	China Population and Employment Statistics Yearbook.	7.74	0.65	6.25	9.65

### 3.3. Measurement and calibration

#### 3.3.1. Result variable measurement

Agricultural development is a fundamental pillar and cornerstone of rural economic progress, and the core of RIR is agricultural development. Rural industries mainly encompass sectors such as plantation, forestry, animal husbandry, and fisheries. Therefore, this study defines RIR in the context of increasing agricultural production, enhancing agricultural value, and boosting agricultural income [46]. Building upon this foundation and in alignment with the digital landscape, this study incorporates the agricultural informatization indicator as a measure of the degree of digitalization within rural industries. Therefore, in this study, the entropy value method is employed to evaluate the level of RIR in 30 provinces of China, aiming to gain a more comprehensive understanding of RIR and facilitate comparisons and rankings of development levels across provinces. For specific descriptions of the outcome variables, please refer to Table 2.

**Table 2:** Result variable description.

Result Variable	Primary Indicators	Secondary indicators	Unit
RIR	increasing agricultural production	Yield per unit area of food crops	Tons/ha
		Production per unit area of cash crops	Ton/ha
	enhancing agricultural value	Value added per capita of primary industry	Yuan/person
		Value added per capita in agriculture	Yuan/person
	boosting agricultural income	Per capita disposable income of rural households	Yuan/person
		Agricultural household savings	Billion yuan
	Agricultural Informatization	Online retail sales of agricultural products	Billion yuan
		Investment in fixed assets in agriculture, forestry, animal husbandry and fishery	Billion yuan
		Average population served by rural postal network	Million people

#### 3.3.2. Conditional variables

1. Digital Infrastructure (DI) encompasses the fundamental components that support the digital economy and social development, encompassing computer networks, communication networks, data centers, and related infrastructure. The measurement of digital infrastructure can be approached through multiple indicators. Therefore, in this study, the entropy method is employed to assess and rank the level of digital infrastructure in 30 provinces of China. The evaluation indicators Number of Domains, Number of Web Pages, Internet broadband access ports, Internet broadband access users, Length of long-distance fiber optic cable lines, Mobile Phone Penetration Rate. For a comprehensive description of these indicators, please consult Table 3.
2. Digital Enterprise (DE) are businesses that leverage digital and information technologies to enhance various aspects of their operations, including management, production, and sales. In this study, the level of digital enterprise development in each province is measured using the proportion of enterprises engaged in e-commerce transactions out of the total number of operating enterprises in that province. Therefore, this indicator serves as an objective measure of the extent of digital enterprise development within a province.
3. Government Support (GS) After reviewing the existing literature, it is apparent that previous studies have mainly focused on measuring for agriculture based on fiscal spending. Therefore, this study employs the ratio of fiscal spending on agriculture, forestry, and water conservancy to the rural population aged six and above, obtained from the 2020 statistical yearbook of each region, as a proxy for government support.
4. Digital Financial Environment (DFE) The degree of development in the digital financial environment can be gauged by the Digital Inclusive Finance Index. This index employs data from the "Peking University Digital Inclusive Finance Index" which is issued by the Peking University Digital Finance Research Center. It serves as a metric to assess the level of development in the digital financial environment across different regions.
5. In terms of Human Resources (HR) level, this study adopts the average years of education as a measurement, based on Fan et al. approach to measuring residents’ digital literacy level [47]. This indicator is calculated by summing the products of the number of individuals at each education level and the corresponding years of education, and then dividing by the total population of rural residents aged six and above. The education levels considered include illiterate, primary, junior high school, high school, secondary vocational education, college education or above, and postgraduate education, with corresponding years of education assigned as 0, 6, 9, 12, 12, 16, and 19, respectively.

**Table 3:** Description of digital infrastructure metrics.

Indicators	Indicator Description
Number of Domains	Indicates the number of domain names owned by the province.
Number of Web Pages	Indicates the total number of pages in the province.
Internet broadband access ports	Indicates the number of Internet access ports in the province.
Internet broadband access users	Indicates the number of Internet users in the province.
Length of long-distance fiber optic cable lines	Indicates the length of long-distance fiber optic cable lines in the province.
Mobile Phone Penetration Rate	Indicates the number of cell phone users in the province as a proportion of the total population.

#### 3.3.3. Data calibration

In the fuzzy set qualitative comparative analysis (fsQCA) method, each of the five conditional variables and the result variable of RIR can be considered as a distinct set, with each case having corresponding degree of membership scores. The calibration process involves assigning membership scores to each case set. In this study, we utilized the direct calibration method [48],which involves three membership points: fully belonging, crossover, and fully not belonging. These points represent values that entirely belong to the target set, do not belong to either the target or non-target sets, and fully do not belong to the target set. To determine the membership points, we employed 0.95, 0.5, and 0.05 as the fully belonging, fully crossover, and fully not belonging points, respectively. The calibration information for the conditional variables and result variable is presented in Table 4.

**Table 4:** Calibration of conditional variables and results.

Conditional and result variables	Calibration		
	fully belonging points (0.95)	fully crossover points (0.5)	fully not belonging points (0.05)
Y: RIR	0.595	0.273	0.120
X1: DI	0.537	0.140	0.036
X2: DE	13.810	10.500	5.635
X3: GS	21.581	4.397	2.908
X4: DFE	412.926	337.717	305.759
X5: HR	8.669	7.797	6.760

## 4. Empirical analysis

### 4.1. Necessary condition analysis

The necessary condition analysis (NCA) method is useful in determining if a set of conditions is necessary in a particular situation and identifying the minimum level of necessary conditions required for a specific outcome based on their effect sizes [49]. Table 5 presents the results of the necessity analysis for the five conditional variables in this study, conducted using R software. Two estimation methods, ceiling regression (CR), and ceiling envelopment (CE) were employed to determine effect sizes. The effect size (d) ranges from 0 to 1, with smaller values indicating a weaker effect. To generate necessary conditions using the NCA method, two conditions must be met: (1) the effect size of the condition variable is greater than or equal to 0.1, and (2) the effect size P-value is significant (P<0.05).

**Table 5:** Results of necessary condition analysis.

Conditions	Method	C-Accuracy	Ceiling zone	Scope	Effect size (d)	P-value
DI	CR	86.7%	0.252	0.91	0.276	0
	CE	100%	0.254	0.91	0.279	0
DE	CR	93.3%	0.101	0.91	0.110	0.08
	CE	100%	0.145	0.91	0.159	0.033
GS	CR	90%	0.122	0.92	0.112	0.054
	CE	100%	0.152	0.92	0.165	0.014
DFE	CR	83.3%	0.275	0.91	0.302	0
	CE	100%	0.270	0.91	0.296	0
HR	CR	90%	0.183	0.94	0.195	0.009
	CE	100%	0.215	0.94	0.228	0.002

Note: 0 ≤ d < 0.1 indicates low level, 0.1 ≤ d < 0.3 indicates moderate level, and d ≥ 0.3 indicates high level. Permutation test (with 10,000 repetitions) was used in the NCA analysis.

Table 5 presents the outcomes of the necessary condition analysis. Notably, digital infrastructure (d value of 0.276 for CR method and 0.279 for CE method), digital financial environment (d value of 0.302 for CR method and 0.296 for CE method), and human resources (d value of 0.195 for CR method and 0.228 for CE method) display significant p-values and moderate effect sizes. As a result, they are regarded as necessary conditions, although not sufficient, for RIR. Conversely, according to the CR method analysis, digitalized enterprises (d value of 0.110) and government support (d value of 0.112) exhibit non-significant p-values. Consequently, they are not deemed necessary conditions for RIR.

Table 6 displays the results of the bottleneck analysis, which indicates the minimum levels of each predictor variable that must be attained to maximize the outcome variable. In order to achieve a 30% level of RIR, a level of 4.2% for digital infrastructure and 6.7% for human resources must be met, while there is no bottleneck level for the other four conditions. To attain a 60% level of RIR, a level of 34.3% for digital infrastructure, 37.6% for digital financial environment, and 24.3% for human resources must be reached, while there is no bottleneck level for the other three conditions.

**Table 6:** NCA necessary condition bottleneck level analysis/%.

RIR	DI	DE	GS	DFE	HR
0	NN	NN	NN	NN	NN
10	NN	NN	NN	NN	NN
20	NN	NN	NN	NN	0.9
30	4.2	NN	NN	NN	6.7
40	14.2	NN	NN	11.4	12.6
50	24.3	NN	NN	24.1	18.5
60	34.3	NN	NN	36.7	24.3
70	44.4	8.0	10.1	49.4	30.2
80	54.4	26.8	29.9	62.1	36.1
90	64.4	45.6	49.7	74.7	41.9
100	74.5	64.3	69.4	87.4	47.8

Note: Effect sizes were calculated using the CR method; NN indicates not necessary.

To gain a deeper understanding of the essential factors influencing the outcomes, this study integrates the NCA method with the fsQCA method to conduct a comprehensive analysis of the necessary conditions and determine whether individual conditions are crucial for RIR. A condition is deemed necessary for the outcome if its consistency exceeds 0.9. Based on the findings presented in Table 7, no necessary conditions have been identified for attaining a high level of RIR. In the context of Necessary Condition Analysis (NCA), necessary conditions imply that X is required for Y at lower levels of X [50]. Therefore, it suggests that lower levels of digital infrastructure, digital financial environment, and human resources are essential for RIR. In this study, we employ QCA to analyze the necessary conditions for achieving a high level of RIR. Necessary conditions in QCA are defined as conditions that meet a certain level of membership. Our findings suggest that while a certain level of digital infrastructure, digital financial environment, and human resources are necessary for RIR, high levels of these factors are not necessary for achieving a high level of RIR. Therefore, we do not identify any necessary conditions for generating high or non-high levels of RIR.

**Table 7:** Necessity analysis of individual conditions.

Condition Variables	High-level RIR		Non-high-level RIR	
	Consistency	Coverage	Consistency	Coverage
High DI	0.837242	0.833687	0.504080	0.568294
Non-High DI	0.566454	0.502205	0.852480	0.855702
High DE	0.685856	0.650708	0.567483	0.609575
Non-high DE	0.588486	0.545814	0.674827	0.708635
High GS	0.611230	0.719064	0.494036	0.658027
Non-high GS	0.709311	0.553215	0.789077	0.696785
High DFE	0.771144	0.828877	0.457627	0.556914
Non-High DFE	0.587775	0.489060	0.859385	0.809580
High HR	0.777541	0.756047	0.605775	0.666897
Non-high HR	0.657427	0.595621	0.778405	0.798455

### 4.2. Sufficiency analysis of high-level rural industry revitalization

The analysis of sufficiency focuses on the perspective of influencing factors. If a specific configuration of multiple conditions always leads to a particular result, then that configuration is a sufficient condition for the result, and the set represented by the configuration is a subset of the result. Typically, a consistency threshold of 0.8 is used to select a standard for sufficiency analysis, although this threshold should not be set lower than 0.7 [51]. In this study, a consistency threshold of 0.8 was used, along with a frequency threshold of 1 for the cases, given that the sample size was small to medium with 30 cases. The consistency threshold for PRI was set at 0.75.

Table 8 displays the findings of the nestedness analysis of simple and intermediate solutions using fsQCA3.0 software. These paths include digital-driven type, digital-government-talent-driven type, digital-enterprise-driven type, and digital-enterprise-talent-driven type. The consistencies of the solutions range from 0.90 to 0.95, and the coverage ranges from 0.41 to 0.48. The four paths exhibit an overall consistency of 0.89, indicating an 89% likelihood of attaining high-level RIR among all configurations that satisfy these paths. The overall coverage is 0.63, indicating that these four paths can explain 63% of the cases of RIR. The empirical analysis has substantial explanatory power, with solutions having a consistency of above 0.80 and coverage having robust explanatory power. Next, we will proceed with a specific analysis and elaboration of each configuration that results in a high level of RIR.

**Table 8:** Analysis of antecedent histories of high level rural industry revitalization.

Conditional Variables	high level RIR			
	Y1	Y2	Y3	Y4
DI	⚫	⚫	⚫	⚫
DE			●	●
GS	⊗	●	⊗	
DFE	⚫	⚫	⚫	⚫
HR	⊗	●		●
Raw Consistency	0.91	0.94	0.90	0.95
Coverage	0.48	0.43	0.45	0.51
Unique Coverage	0.03	0.01	0.00	0.00
Solution consistency	0.89			
Solution coverage	0.63			

Note: ⚫means the core condition exists, ●means the edge condition exists, ⊗ means the core condition is missing, ⊗ means the edge condition is missing, and space means both are missing.

#### (1) Digitaldriven

Based on the Y1 configuration, attaining high-level RIR necessitates improvements in digital infrastructure and the digital financial environment. Strengthening digital infrastructure can expand and deepen information accessibility, allowing rural enterprises to efficiently and intelligently access information from both upstream and downstream sources and reach bottom-level consumers. Concurrently, a robust digital financial environment offers rural enterprises greater opportunities for financing and investment, thus further stimulating rural industrial development and fostering innovation. The enhancement of these two conditions will unlock additional development opportunities and potential for rural industries. The Y1 configuration can explain cases in Anhui, Fujian, Jiangxi, Shandong, and Henan. For example, Henan Province has made significant efforts to advance the development of digital infrastructure, including constructing 12.47 million 5G base stations, resulting in complete 5G network coverage in rural hotspots. In addition, the government in Henan is actively promoting digital inclusive financial services, and the digital economy has grown to reach 1.6 trillion yuan. The ongoing enhancement of digital infrastructure and the digital financial environment has facilitated the integration of rural IoT, which has created new opportunities for digital rural industries.

#### (2) digital-government-talentdriven

Building upon the Y1 configuration, two additional edge conditions, namely government support and human resources, play vital roles in contributing to RIR. Together with improved digital infrastructure and a favorable digital financial environment, these conditions form a driving pathway for promoting RIR. Government support assumes a crucial role in various aspects, encompassing policy formulation, financial funding, and tax incentives, providing robust guarantees for rural industrial development. Moreover, the government establishes platforms for rural industrial growth, fostering industry integration and coordinated progress. Additionally, the government’s supervision and services ensure the legitimate rights and stable development of rural industries. Regarding human resources, the support of highly skilled talents proves indispensable for RIR. The government can enhance the quality of farmers through training and educational programs, elevating their skills and knowledge to provide robust talent support for industrial development. Furthermore, implementing relevant policies to attract external talents to venture into rural areas not only enriches the talent pool but also facilitates the exchange and inheritance of experience and technology. In Zhejiang Province, for example, rural industry development is heavily reliant on significant financial investment from the government. In 2020, the Zhejiang provincial government invested 37.86 billion yuan in rural informatization funds. With government guidance, the Digital Economy Talent Training and Evaluation (Ningbo) Base was established to meet the talent requirements of the digital age.

#### (3) digital-enterprisedriven

In the Y3 configuration, the presence of robust digital infrastructure and a sound digital financial environment emerges as crucial factors for RIR. Meanwhile, digital enterprises assume a supportive role, and government support is not deemed a necessary condition for RIR. This suggests that with strong digital infrastructure and a well-established digital financial environment, the development of digital enterprises can still drive RIR even in the absence of financial support from local governments. Sufficient digital infrastructure and a sound digital financial environment provide essential support for rural enterprises, facilitating information acquisition, financing, and investment, thus propelling rural industrial development and innovation. It is worth emphasizing that digital enterprises, as the primary organizational form, act as a significant driving force behind RIR, thanks to their robust financial capabilities and technological expertise. Moreover, the organizational coordination ability of digital enterprises fosters the establishment of industrial and value chains, creating a complete rural industrial ecosystem. Furthermore, through the application of advanced digital technologies and management practices, digital enterprises expedite the digitization and intelligence of rural industries, enhancing production efficiency and product quality. In Shandong Province, the growth of digital-related enterprises has been remarkable, providing robust support for the digital transformation of rural industries in the region. Shandong boasts more than 126,000 digital-related enterprises, the second-highest number in the country. These enterprises have digitized and made intelligent various links in agricultural production, processing, and sal es, thus enhancing production efficiency and quality. The development of digital enterprises has also spurred agricultural technological innovation and rural talent training, augmenting the overall competitiveness and innovation capacity of rural industries in Shandong Province.

#### (4) digital-enterprise-talentdriven

Building on the Y3 configuration, the incorporation of human resources becomes essential. Rural areas encounter challenges in attracting talent, as highly skilled individuals often prefer to remain in urban centers, resulting in a shortage of talents in rural regions. However, the development of digital enterprises typically necessitates a considerable number of technical professionals, presenting an opportunity for rural areas to entice talent back. The growth of digital enterprises not only offers employment opportunities and developmental platforms for human resources but also relies on exceptional talent as a critical support for their own progress. These highly skilled individuals play pivotal roles within digital enterprises, being responsible for tasks such as technological research and development, innovative design, market expansion, and other crucial areas. By tapping into the potential of human resources and luring talent to return to rural areas, the advancement of digital enterprises contributes not only to RIR but also to the overall development of rural communities. For instance, Guangdong, as an economically developed province, possesses a relatively complete digital infrastructure and digital financial environment, and has the highest number of digital enterprises in the country. This lays a solid foundation and provides support for the digital transformation and development of rural industries, as well as digital solutions and technical support. Moreover, Guangdong has been consistently strengthening the development of rural talent and has implemented policies such as the "Work Plan for Rural Revitalization Assistance by Stationed Personnel in Guangdong Province." This involves sending technology experts to assist in rural revitalization and selecting agricultural science and technology personnel to be stationed in rural areas to provide targeted assistance.

### 4.3. Robustness analysis

There are various methods for conducting robustness tests in fsQCA research. When the results of the robustness test have a subset relationship with the original results, it indicates that the results are robust. In this study, robustness tests were conducted by changing the consistency threshold to 0.85 and 0.75, respectively. The consistency threshold was also increased from 0.8 to 0.85. The results of both analyses were consistent with the original results, confirming the reliability of the research findings.

### 4.4. Analysis of regional heterogeneity paths of industrial revitalization in eastern, central and western China’s villages

The vast expanse of China’s territory and the diverse distribution of economic development and resources lead to significant regional disparities in the development of the digital economy, technological infrastructure, and organizational applications. In addition, the institutional environment in various regions can significantly influence the development of rural industries. To tackle this problem, this research divides the sample data into three major economic zones in accordance with China’s "Seventh Five-Year Plan" (1986–1990): eastern, central, and western China. Through a comparison and analysis of the effects of technology, organization, and environmental conditions on RIR in each region, this study aims to examine the differences in the impact of RIR across different regions, providing valuable insights for policy formulation. The findings of this study are presented in Table 9. The eastern region demonstrates configuration paths E1, E2, E3, and E4, while the central region adheres to path M1, and the western region encompasses paths W1 and W2. These variations in configuration paths for RIR across different regions of China are clearly evident, emphasizing the significant regional disparities.

**Table 9:** Analysis of antecedent histories of high-level RIR paths in East, Central and West.

Conditional Variables	Eastern Region				Central Region	Western Region	
	E1	E2	E3	E4	M5	W6	W7
DI		●	⊗	●	⚫	⚫	⚫
DE	⊗	⚫	⚫	⚫	●	⚫	⚫
GS	●	⊗	⊗	●	⊗	⊗	
DFE	⚫	⊗	●	●	●	⚫	⚫
HR	●	⊗	⊗	⊗	⊗		●
Consistency	0.95	0.87	0.91	0.99	0.89	0.94	0.98
Coverage	0.38	0.33	0.21	0.30	0.43	0.59	0.53
Unique Coverage	0.03	0.01	0.00	0.00	0.43	0.18	0.12
Solution consistency	0.90				0.89	0.95	
Solution coverage	0.58				0.43	0.71	

Note: ⚫means the core condition exists, ●means the edge condition exists, ⊗ means the core condition is missing, ⊗ means the edge condition is missing, and space means both are missing.

In the eastern region, path E1 is characterized by the digital financial environment as its core condition, whereas the absence of digital enterprises serves as its core absence condition. Paths E2 to E4, on the other hand, share the core condition of DE and the core absence condition of human resources. The developed economy and high level of digitalization in the eastern regions of China allow for the promotion of RIR, even in the absence of digital enterprises. This is made possible through a combination of government support and improvements to the digital financial environment; Digital enterprises play a critical role in the RIR, facilitating their development even in the absence of government support and human resources. Digital enterprises play a pivotal role in facilitating the growth of rural industries by providing access to state-of-the-art digital technology and robust network platforms that connect them with markets. Additionally, with support from digital finance, these enterprises offer specialized technology and industry chains, enabling rural industries to upgrade their operations and increase their competitiveness. Hence, policymakers in the eastern region ought to augment policy support and promote the guidance and encouragement of more enterprises to invest in the advancement of digital rural industries.

In the central region, path M1 reveals that the region lags behind the eastern region in terms of economic development, digitalization, and talent attraction. Despite this, achieving high-level RIR is still feasible through other crucial factors, even without government support and human resources. In the central region, digital enterprises and the digital financial environment are not essential conditions for RIR, implying significant room for growth and development in these aspects. digital infrastructure is a core existing condition in the central region, indicating that the region has a solid foundation in DI, which provides crucial support for the growth and development of digital enterprises and digital finance. In conclusion, the central region should augment investment and policy support in digital enterprises and the digital financial environment, and capitalize on the benefits of digital infrastructure to boost digitalization and talent acquisition. This approach can foster economic development and industrial upgrading.

In the western region, there are two high-level (W1, W2) configuration paths for RIR, both of which require DI, DE, and DFEs. Due to harsh natural conditions, sparse population, poor transportation, and backward agricultural technology, rural economic development in the western region faces many challenges. Therefore, improving DI is essential for promoting RIR. The western region is characterized by economic underdevelopment and a relative lack of digital technology. Additionally, due to its vast size and sparse population, the region experiences significant talent loss, which further impedes the development of rural industries. The Chinese government has been addressing these challenges by gradually increasing investments in DI, aimed at promoting the development of the digital economy and creating a new pattern of western development. When promoting the RIR, it is crucial to take into account the conditions in the economically under developed western region. Therefore, increasing investment in DI and digital enterprise development, as well as providing stronger supervision and support for the DFE, is essential to address these challenges. Encouraging all parties to actively participate in the development of the digital economy is also crucial. These measures can effectively promote the RIR in the western region and facilitate sustainable development of the rural economy.

## 5. Discussion

### 5.1. On combinatorial factors of RIR

It can be observed that the core conditions from Configuration 1 to Configuration 4 all involve digital infrastructure and the digital financial environment, both of which play a universal role in achieving high-level RIR. The digital financial environment is closely related to the level of digital infrastructure, and the two mutually influence each other. The development of digital infrastructure provides the conditions for the modernization transformation of rural industries and lays the foundation for the development of the digital financial environment [52]. With improved infrastructure and a favorable financial environment, the circulation of capital in rural industries is enhanced, while difficulties in funding and financing channels are reduced, promoting equal opportunities [53]. As digitalization advances, more and more countries prioritize internet broadband and other digital infrastructure. However, compared to urban areas, rural digital development still faces limitations. Most rural areas lack sufficient internet infrastructure, experience unstable power supply, and lag behind in digital financial and e-commerce development [54]. Additionally, a majority of rural residents lack basic financial and internet knowledge, which hampers their participation in inclusive digital finance. Some empirical studies have found that rural residents with limited access to internet infrastructure do not receive effective stimulation for their financial needs, leading to further difficulties in their lives [55]. Therefore, both the government and businesses need to make joint efforts, increase investments and development in rural digital infrastructure and the digital financial environment, enhance the digital literacy of rural residents, and thus promote RIR.

Human resources, as a core prerequisite for innovation, play a crucial role, as well-educated talents actively participate in innovative activities [56]. High-level human capital enables individuals to adapt to rapidly changing environments, continuously learn, improve, and effectively absorb and apply knowledge for innovation [57]. In Configurations Y2 and Y4, human resources are combined with government support and digital enterprises, illustrating that human capital is influenced by organizational conditions and requires formal and informal institutional support. In urban areas, the government provides substantial policy subsidies and support to attract talents, while also drawing numerous research institutions and corporate research centers, offering a broader development platform for highly qualified individuals [58]. In contrast, rural areas have relatively limited technological resources, struggling to provide a comparable innovation environment, which leads to a preference for highly skilled talents to stay in cities [5]. Therefore, it is imperative for rural areas to increase investments and support from the government and businesses in RIR, providing more development opportunities for talents to promote the vigorous development of rural industries.

In rural development, the government plays a pivotal role; however, research indicates that government-led rural construction has certain drawbacks that can negatively impact rural development [59]. As such, it is essential for businesses to assume more significant social responsibilities in this context [60]. An excess of government assistance may lead to rural areas becoming overly reliant on the government, thereby limiting their potential for independent development [61]. Nonetheless, this limitation can be mitigated by encouraging the involvement of market capital, which can supplement the government’s shortcomings in rural construction. By attracting more market capital to invest in rural development under government funding support, a more balanced and sustainable development model can be achieved [9, 62].The findings of Configuration Y3 underscore the significant role of businesses in rural development. To alleviate the government’s financial burden and avoid the negative consequences of excessive reliance on government assistance, it is essential for the government to actively encourage market capital to invest in rural development. Moreover, establishing cooperation platforms to promote technology exchange and innovation collaboration can enhance the innovation capacity and competitiveness of small and medium-sized enterprises. Through the implementation of these measures, the participation of enterprise capital will infuse new momentum into RIR.

### 5.2. Contributions

This study has made several significant contributions to the field of RIR in China. Firstly, unlike many previous studies that focused on exploring the net effects of various potential factors on rural industrial development, this research goes beyond the net effect approach [63]. It recognizes the complexity of the RIR process, which involves the intricate interaction of multiple factors. By uncovering the interaction of technology, organization, and environmental conditions to form path configurations, the study reveals that these configurations lead to achieving the same desired outcomes through "different but converging" pathways [51]. This finding deepens researchers’ understanding of the complex mechanisms underlying rural industrial development.

By employing a combination of fsQCA and NCA methods, we have effectively identified and distinguished the sufficiency and necessity causal relationships in the context of RIR. Unlike traditional regression analysis, which primarily identifies sufficient conditions for outcomes, this research incorporates necessary analysis to identify the essential factors determining the outcomes. The integration of both approaches was advocated by Fainshmidt, the editor-in-chief of the International Business Journal, as a means to explore complex causal relationships. Therefore, this paper contributes to the advancement of research on the necessary and sufficient relationships among the influencing factors of RIR in the context of the digital economy.

In addition to conducting a configurational analysis at the national level, this study also takes into account geographical and economic differences to analyze the diverse pathways of rural industrial development in the eastern, central, and western regions of China. This provides valuable practical implications for policymakers in regions at different stages of development. The universality demonstrated by digital infrastructure and the digital financial environment at the national level highlights the critical significance of developing digital rural areas for industrial growth in the current digital economy context. However, the relatively superior economic development in the eastern region compared to the western region, along with its more advanced digital infrastructure and digital financial environment, necessitates a greater focus on other factors to achieve RIR [64]. This is helpful for local governments to select targeted development models for RIR based on local conditions.

### 5.3. Policy implications

Commencing with path configurations, the "digitaldriven" trajectory necessitates a focus on enhancing digital rural infrastructure and digital finance to propel rural digitalization. Concurrently, harnessing digital financial platforms can facilitate the attainment of RIR. By fostering the digital advancement of rural industries within the context of digitalization, significant opportunities and revitalization prospects will undoubtedly ensue for rural industrial development. In the "digital-government-talentdriven" configuration path, the government should augment investments in rural education, thereby enhancing the learning environment and educational resources available to rural youth. Moreover, implementing pertinent policies to incentivize high-quality talents to participate in entrepreneurial activities and seek employment opportunities in rural areas is essential. Furthermore, the integration of top-notch research teams and skilled professionals into rural regions can foster the convergence of technological innovation and industrial development. In the "digital-enterprisedriven" configuration path, the emphasis should be on the pivotal role of digital enterprises, harnessing their digital technologies and innovative management practices to propel the digital transformation of rural industries. In the "digital-enterprise-talentdriven" configuration path, the leadership role of digital enterprises should be fully harnessed, relying on the support of high-quality talents to propel the development of rural industries. Emphasizing talent recruitment and development is essential, as high-quality talent plays a pivotal role in the growth of digital enterprises. Thus, enterprises must bolster their efforts in talent cultivation and recruitment to attract exceptional individuals and contribute to the RIR.

From the perspectives of different regions, the analysis of China’s eastern, central, and western regions reveals significant disparities in their paths toward RIR. Consequently, local governments should consider their respective economic development levels and resource endowments when determining strategies for RIR. In the eastern region, leveraging its existing advantages, there should be encouragement for more enterprises to invest in the development of rural digital industries. For the central region, the focus should be on enhancing the construction of digital financial environments to provide rural enterprises with increased opportunities for financing and investment. Additionally, utilizing the existing digital infrastructure can facilitate rural enterprises in accessing market information more intelligently and conveniently, thereby promoting their digital transformation and upgrading. As for the western region, which is at a relatively backward stage, increased investment in digital infrastructure and the development of digital enterprises is essential to bridge the digital development gap. Strengthening supervision and support for digital finance is crucial to ensure the stable development of the digital financial environment. Simultaneously, encouraging active participation from social organizations, governments, and enterprises in rural digital development can effectively implement China’s Western Development Strategy.

## 6. Conclusions

This article applies the TOE framework and employs the fsQCA method to analyze the path of grouping in 30 Chinese provinces (excluding Hong Kong, Macao, Taiwan, and Tibet due to missing data). The study explores the "joint effect" of different groupings formed by five factors on the RIR and analyzes the key paths affecting the RIR through multiple combinations. The main research conclusions are as follows: first,from an individual variable standpoint, none of the five influencing factors can be deemed a necessary condition for achieving high-level RIR. Second, based on distinct combinations of core conditions, the paths to attaining high-level RIR can be categorized into four types: digital-driven, digital-government-talent-driven, digital-enterprise-driven, and digital-enterprise-talent-driven. These four configuration paths signify that high-level RIR emerges from the collaborative efforts of multiple factors, all aiming towards the common objective of revitalization. Among these paths, digital infrastructure and digital financial environment serve as core conditions found in all four, highlighting their universal role in facilitating high-level RIR. Third, significant disparities exist in the driving paths of RIR across the eastern, central, and western regions of China. Within the eastern region, the path led by organization primarily hinges on digital enterprises serving as the core condition. In the central region, the path led by technology primarily relies on digital infrastructure as the core condition. As for the western region, the path led by technology-organization-environment primarily depends on digital infrastructure, digital enterprises, and digital financial environment as the core conditions. These discernible variations in paths across different regions further elucidate the diverse conditions contributing to the varying levels of high-level RIR.

This study has several limitations that warrant consideration. Firstly, the dataset used in this research is limited to data from a single year, and it lacks cross-year case data. Consequently, the temporal dimension of the research findings may be constrained in terms of explanatory power. Future studies could enhance the robustness of the research by incorporating multi-year data for dynamic quantitative analysis.Secondly, the structural comparison method of fsQCA serves as a methodological bridge between qualitative and quantitative approaches, providing a systematic framework to examine the complex causality within cases and explore the interrelationships among cause-related variables. However, it is imperative to acknowledge that the method is still evolving, and it has faced critique regarding data calibration and the potential reduction of nuanced insights and richness inherent in qualitative data. Therefore, future scholars should diligently address and optimize these controversies to advance the application and effectiveness of the fsQCA method in research endeavors.
